# Generating Novel Male Sterile Tomatoes by Editing Respiratory Burst Oxidase Homolog Genes

**DOI:** 10.3389/fpls.2021.817101

**Published:** 2022-01-10

**Authors:** Xiaojuan Dai, Huanan Han, Wei Huang, Lianghui Zhao, Minglei Song, Xuesong Cao, Chenglan Liu, Xiaomu Niu, Zhaobo Lang, Changle Ma, Hongtao Xie

**Affiliations:** ^1^College of Life Sciences, Shandong Normal University, Jinan, China; ^2^BellaGen Biotechnology Co., Ltd., Jinan, China; ^3^Shandong Plant Protection Station, Jinan, China; ^4^National Key Laboratory of Plant Molecular Genetics, Shanghai Center for Plant Stress Biology, Center for Excellence in Molecular Plant Sciences, Chinese Academy of Sciences, Shanghai, China

**Keywords:** male sterile, tomato, CRISPR, RBOH, hybridization breeding

## Abstract

Hybrid breeding of tomatoes (*Solanum lycopersicum*), an important vegetable crop, is an effective way to improve yield and enhance disease and stress resistance. However, the efficiency of tomato hybridization is hindered by self-fertilization, which can be overcome using male sterile lines. It has been reported that reactive oxygen species (ROS) act as a key regulator for anther development, mediated by *RBOH* (*Respiratory Burst Oxidase Homolog*) genes. Here, two tomato anther-expressed genes, *LeRBOH* (Solyc01g099620) and *LeRBOHE* (Solyc07g042460), were selected to cultivate novel tomato male sterile strains. By using a CRISPR/Cas9 system with a two-sgRNA module, the *lerboh*, *lerbohe*, and *lerboh lerbohe* mutant lines were generated, among which the *lerbohe* and *lerboh lerbohe* mutants displayed complete male sterility but could accept wild-type pollens and produce fruits normally. Further analysis uncovered significantly decreased ROS levels and abnormal programmed cell death in *lerboh lerbohe* anthers, indicating a key role of ROS metabolism in tomato pollen development. Taken together, our work demonstrates a successful application of gene editing via CRISPR/Cas9 in generating male sterile tomatoes and afforded helpful information for understanding how *RBOH* genes regulating tomato reproduction process.

## Introduction

Tomato (*Solanum lycopersicum*) is a highly cultivated and consumed vegetable around the world. Improvements in tomato crops are largely dependent on hybrid breeding, which can efficiently increase yields or enhance biotic/abiotic resistance (Tamta and Singh, 2017; Kim and Zhang, 2018). However, because tomato can self-fertilize, hybrid breeding requires excessive time and labor for artificial emasculation. Thus, tomato male sterile germplasms are critical for efficient hybrid breeding (Kim and Zhang, 2018). Nearly 50 male sterile mutants have been reported (Gorman et al., 1997; Sawhney, 2004; Cheema and Dhaliwal, 2005; Gorguet et al., 2009; Zhang et al., 2016; Pucci et al., 2017; Cao et al., 2019; Liu et al., 2019; Yu et al., 2020), but most strains possess undesirable phenotypes. Some sterile lines can still perform some self-crossing as they still produce a few active pollens. In some functional sterile strains, the stigmas are shorter than the anther tube, which effectively prevents natural self-crossing but makes artificial pollination very difficult (Perez-Prat and Campagne, 2002). In addition, in a strain with completely abortive pollens, the pistils are also anomalous, resulting in a low outcrossing rate (Chen et al., 2004). Thus, novel male sterile germplasms overcoming these disadvantages are still urgently needed.

Among the many factors affecting the tomato reproduction process, just as temperature and light (Li et al., 2006; Fu et al., 2014; Zhu et al., 2015; Shen et al., 2019), ROS (Reactive Oxygen Species) is also proved as a key regulator of anther development and thus pollen fertility (Luo et al., 2013). The transcription factor MADS3 up-regulates *MT-1-4b*, a ROS scavenging protein, and thus negatively regulates ROS content in rice anthers, influencing the maturation of pollens (Hu et al., 2011). As a necessary process for normal pollen maturation, the programmed cell death (PCD) of the tapetum is associated with ROS metabolism (Joanna, 2003). In rice, the mitochondrial protein Wa352 inhibits the ROS scavenging function of COX11, promoting PCD in the tapetum and subsequently leading to pollen abortion (Luo et al., 2013; Wang et al., 2018). Another rice male sterile mutant *dtc1* also exhibits decreased ROS accumulation in the anthers and a delayed PCD in hypertrophic tapetum (Yi et al., 2016). In *Arabidopsis*, reduced ROS in the tapetum results in delayed PCD, resulting in decreased pollen fertility (Xie et al., 2014). Similarly, increasing ROS accumulation caused by Brassinolides (BRs) was found to promote degradation of the tapetum in tomatoes (Yan et al., 2020). Together, these reports confirm the important role of ROS in tapetum development, but the regulatory mechanism of ROS accumulation seems rather complex in general (Ullah and Yi, 2019). Thus, it is necessary to analyze genes relating to ROS metabolism in anthers.

As a membrane protein that converts NADPH to O^2–^ and electrons, RBOH (Respiratory Burst Oxidase Homolog) members are important resources of ROS (Chapman et al., 2019). As described in our previous work, *AtRBOHE* plays a key role in regulating ROS accumulation in the anthers and subsequently mediates development of the tapetum in *Arabidopsis* (Xie et al., 2014). In tomatoes, knock-down of *LeRBOH1* via RNAi (RNA interference) reduces ROS content and influences PCD in the tapetum, resulting in an about 40% decrease in seed number (Yan et al., 2020). However, as a protein family with many homologous members, the function of other tomato RBOHs during anther development is still unclear, and whether a practical male sterile germplasm can be generated by modulating RBOH genes is still uncertain. In tomatoes, there are in total 8 *RBOH* genes (*LeRBOHA*, *LeRBOHB*, *LeRBOHD*, *LeRBOH*, *LeRBOHE*, *LeRBOHH*, *LeRBOH1*, and *LeWif1*), among which *LeRBOHE* and *LeRBOH* show a temporal expression pattern during anther development (Yu et al., 2017). As the qPCR results describing, *LeRBOH* displayed a anther-specific high expression while *LeRBOHE* exhibited the highest expression in anther and stem, indicating they may play a role in regulating anther development. However, whether these two *RBOH* genes regulate pollen fertility remains unknown.

In certain cultivars, mutation of key fertility genes via CRISPR can be an efficient alternative to the occasionally used, time-consuming process of obtaining male sterile lines by screening natural mutants (Du et al., 2020). Here, relying on a double-sgRNA module, we edited *LeRBOHE* and/or *LeRBOH* with SpCas9 in the commercial tomato variety Alisa Craig (AC). We observed that *LeRBOHE* and *LeRBOH* played a predominate and minor roles, respectively, in mediating tomato pollen maturation, concomitant with defects in ROS synthesis. Our work revealed mechanism by which *RBOHs* regulate development of tomato anthers, and successfully established a complete male sterile strain for tomato hybrid breeding.

## Materials and Methods

### Plant Materials and Growth Conditions

Tomato (*Lycopersicon esculentum*, Alisa Craig) were grown in a climate chamber at 25/20°C (day/night) under long days (16 h day and 8 h night).

### Plasmid Construction

*LeRBOH* (Solyc01g099620) and *LeRBOHE* (Solyc07g042460) gene loci were retrieved from the tomato website.^1^ sgRNAs for the CRISPR/Cas9 target sites of *LeRBOH* and *LeRBOHE* were designed with http://skl.scau.edu.cn/targetdesign// (sgRNA1: TAGCTAGCAAGCTCGAAAAG; sgRNA2: TCTAGCAAGTAA TCCGTCTT). Using the tRNA-scaffold as a template, the fragments were amplified with gLeRBOHE-F/R primers respectively, and were transferred into the CRISPR/Cas9 vector after digestion with Bsa I. All primers used in the study are listed in Supplementary Table 1.

### Mutant Detection

The genomic DNA of the transgenic plants was extracted from leaves via the CTAB method (Porebski et al., 1997). Hyg-F/R, Cas9-F/R, and M13-F/gRNA-R primers were used to detect the hygromycin resistance gene, Cas9 and the sgRNA, respectively. LeRBOH-F/R and LeRBOHE-F/R were used for identification of edited targets.

### Characterization of the Mutant Phenotype

The morphology of the plant, pistil, stamen, and fruit were photographed with a digital camera (Nikon, D750). Seed numbers, seed generating ratios, leaf numbers and plant heights of AC lines and mutants were analyzed by Student’s *t*-test. The mature pollen was dyed with Alexander (Solarbio, Beijing, China) as described previously (Peng et al., 2013), and photographed with a Leica microscope (DMLB, Germany).

### Observation of Pollen Morphology

Anther morphology was observed using a scanning electron microscope (Leica, DM6B, Germany). Open flowers in good growth condition were taken. The petals were removed, and half of the anthers were torn off and gently stuck onto a metal stage with double-sided tape. For pollen observation, wild-type and mutant pollens were soaked in 2.5% glutaraldehyde fixative solution, and fixed at 4°C for 24 h. After rinsing 3 times with phosphate buffer solution, and dehydrating through successive alcohol gradients, the pollens were placed in isoamyl acetate and dried at the critical point of CO_2_. Finally, the samples were photographed by scanning electron microscope (HITACHI, TM3030) after vacuum coating.

### TUNEL Assay

Paraffin-embedded anthers at different developmental stages were collected for processing. *In situ* nick-end labeling of nuclear DNA fragmentation was performed with the Dead End Fluorometric TUNEL system according to the supplier’s instructions (Promega, G3250). Samples were analyzed with a Leica DM6B microscope using a 488-nm/510-nm excitation/emission spectrum for fluorescein and a 530-nm/640-nm excitation/emission spectrum for propidium iodide as previously described (Phan et al., 2011).

### Histology of Anthers and Histochemical Assays for Reactive Oxygen Species

Anthers at different developmental stages were fixed and then embedded in paraffin. Sections (8 μm) were cut using a Leica Histocore MULTICUT microtome, stained with ruthenium red, and photographed with a Leica (DM6B, Germany) microscope in bright field. To visualize levels of the superoxide anion as a measure of ROS content, anthers of different developmental stages were stained with 0.5 mM NBT (Sigma, N5514).

### DAPI Staining

Pollens of different developmental stages were placed in 1 mg/L DAPI (Aladdin, D106471) solution and placed in a dark, moisturizing environment at 60°C to stain the microspores. The number of nuclei at each stage of microspore development was observed under a fluorescence microscope (DMLB, Leica, Germany).

## Results

### Mutation of *LeRBOH/LeRBOHE* via CRISPR/Cas9

**FIGURE 1:**
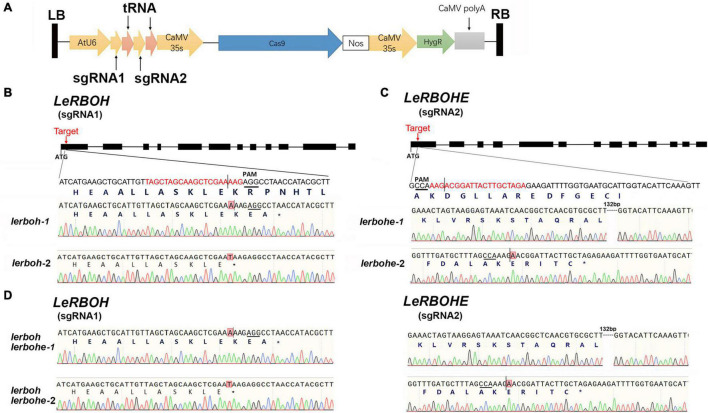
Construction of *lerboh/lerbohe* via CRISPR/Cas9. **(A)** Structure of T-DNA insertion containing double sgRNAs for *LeRBOH* and *LeRBOHE*, Cas9 driven by the CaMV35S promoter, and *HygR* (hygromycin resistance gene) as a resistance marker. **(B–D)** Mutations in the *LeRBOH* or *LeRBOHE* locus in *lerboh* **(B)**, *lerbohe* **(C)**, and *lerboh lerbohe* **(D)** mutants. Black squares indicate exons while line segments indicate introns in the sketch maps of the *LeRBOH/LeRBOHE* genes (top). The protospacer is marked as red while the PAM is underlined. In the Sanger sequencing results, short vertical lines indicate cleavage sites by Cas9 in the *LeRBOH/LeRBOH* genes. Inserted bases are highlighted. The dotted lines represent deletions in the mutants. * means stop condon.

It has been reported that *LeRBOHE* and *LeRBOH* show a temporal expression pattern during anther development (Yu et al., 2017). To analyze the function of *LeRBOH* and *LeRBOHE* during anther development, we generated tomato mutants via the CRISPR/Cas9 system. Two sgRNAs, with spacer sequences specifically targeting the first exon of *LeRBOH* and *LeRBOHE*, respectively, were separated by tRNA and driven by the *Arabidopsis* U6 promoter, while SpCas9 was driven by a CaMV35S promoter (Figure 1A). The modules were constructed into the T-DNA containing plasmid and transformed into the AC strain via *Agrobacterium tumefaciens*. Eight T-DNA-free mutants were identified in the T_1_ lines (Supplementary Figure 1). Among these mutants, there was a homozygous double knockout mutant (*lerboh lerbohe-1*) with an “A” insertion in the *LeRBOH* gene locus (induced an early “TAA”), and a 132-bp deletion (caused a 44-amino-acid deletion in the NADPH-Ox domain of LeRBOHE) in the *LeRBOHE* locus (Figure 1D and Supplementary Figure 2). As coproducts, two T-DNA-free lines containing the same mutation in either *LeRBOH* (*lerboh-1*) or *LeRBOHE* (*lerbohe-1*) as *lerboh lerbohe-1* were also selected, respectively (Figures 1B,C). In addition, we also obtained a mutant line with a “T” insertion in the first exon of *LeRBOH* (*lerboh-2*), generating an early “TAA,” a mutant line with a “A” insertion in the first exon of *LeRBOHE* (*lerbohe-2*), resulting in premature termination by an early “TAG,” and another double mutant (*lerboh lerbohe-2*) with the same *LeRBOH* and *LeRBOHE* mutations as *lerboh-2* and *lerbohe-2*, respectively (Figures 1B–D).

### *lerbohe* and *lerboh lerbohe* Mutants Caused Absolute Male Sterility

**FIGURE 2:**
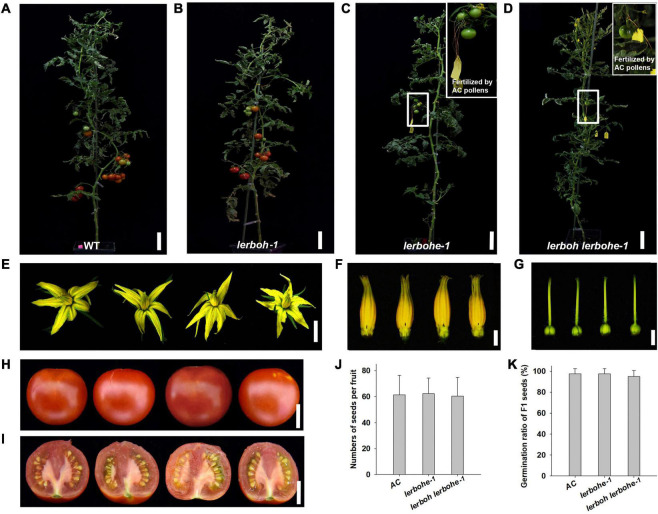
Developmental phenotypic analysis of *lerboh/lerbohe* mutants. **(A–D)** Growth phenotype of WT (AC) **(A)**, *lerboh-1* **(B)**, *lerbohe-1* **(C)**, and *lerboh lerbohe-1* **(D)** plants. The cross fruits fertilized by AC pollens in *lerbohe-1* or *lerboh lerbohe-1* lines are boxed and shown enlarged in the inset. **(E–G)** Phenotypic analysis of flowers **(E)**, stamens **(F)**, and Pistils **(G)** for (from left to right) WT (AC), *lerboh-1*, *lerbohe-1*, and *lerboh lerbohe-1.* **(H,I)** Fruit phenotype of (from left to right) AC, *lerboh-1* (self-fertilized) and *lerbohe-1*, *lerboh lerbohe-1* (fertilized by AC pollens). **(J)** Number of seeds in a fruit of AC (self-fertilized) and *lerbohe-1*, *lerboh lerbohe-1* (fertilized by AC pollens). **(K)** Germination ratio of F_1_ seeds. Scale bar: plants, 10 cm; flowers, 1 cm; flower organs, 3 mm; fruits, 2 cm.

To analyze the role of *LeRBOH* and *LeRBOHE* in tomato male fertility, *lerboh-1*, *lerbohe-1*, *lerboh lerbohe-1*, and AC lines were cultivated together at suitable conditions for their life-cycle (Figure 2). Amazingly, both *lerbohe-1* and *lerboh lerbohe-1* lines could not fruit at all, while fruits were produced normally in *lerboh-1* (Figures 2A–D). And interestingly, there were no obvious visible differences in either vegetative growth or flower development between the mutants and AC. Both *lerbohe-1* and *lerboh lerbohe-1* mutants displayed nearly normal growth morphology as the AC stains, including similar plant heights and numbers of compound leaves on main stem (Table 1). After growing under normal condition in greenhouse for 80 days, there were no obvious difference in both flower numbers and flower morphology between mutant and AC lines (Table 1 and Figures 2E–G). Furthermore, as the cross-pollination assay showed, when wild-type pollens were stuck to the stigma, *lerbohe-1* and *lerboh lerbohe-1* could fruit normally (Figures 2C,D). The hybrid fruits became ripe with ordinary morphology (Figures 2H,I), full of normal seeds (with similar seed numbers as the AC fruits) with a good germination ratio (Figures 2J,K). However, no fruits appeared when the mutated pollens were stuck to the stigma of emasculated AC (Supplementary Figure 3). In addition, *lerboh-2*, *lerbohe-2*, and *lerboh lerbohe-2* exhibited the same phenotypes as *lerboh-1*, *lerbohe-1*, and *lerboh lerbohe-1*, respectively (Supplementary Figure 4). Thus, we acquired near-ideal tomato male sterile strains with no obviously undesirable side effects by mutating two *RBOH* genes, with the effects driven largely by *LeRBOHE*.

**TABLE 1:** Growth phenotypic analysis of *lerboh/lerbohe* mutants.

	AC	*lerboh-1*	*lerbohe-1*	*lerboh lerbohe-1*
Number of compound leaves	26 ± 3	26 ± 3	27 ± 4	27 ± 3
Number of flower clusters	8 ± 2	8 ± 2	9 ± 2	9 ± 2
Flower number per cluster	11 ± 3	11 ± 3	11 ± 3	11 ± 3
Plant height (cm)	164.3 ± 7.0	163.7 ± 6.0	169.7 ± 8.5	165.0 ± 7.1

### *lerbohe* and *lerboh lerbohe* Mutants Displayed Abnormal Pollen Development

**FIGURE 3:**
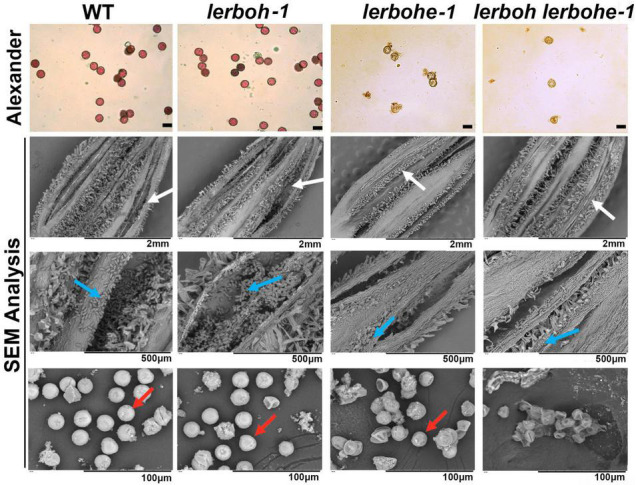
Analysis of pollen morphology and activity in AC, *lerboh-1*, *lerbohe-1*, and *lerboh lerbohe-1* lines. First line, Alexander stain of pollens; second to fourth lines, SEM detection for anthers and pollens. White arrow, anther cracking; blue arrow, pollens; red arrow, pollens with normal morphology. Scale bar in the first line: 20 μm.

The cross-pollination assays suggested that the *lerbohe* or *lerboh lerbohe* mutants produced abnormal pollens. To confirm this hypothesis, pollens of *lerboh-1*, *lerbohe-1*, *lerboh lerbohe-1*, and AC lines were selected and dyed with Alexander to visualize pollen activity (Figure 3). There were much fewer pollens spread from anthers of two male sterile mutant lines (*lerbohe-1*, *lerboh lerbohe-1*) than wild type or *lerboh* ones. And as expected, wild-type and *lerboh* pollens were active, appearing strongly stained, round and full, while nearly all *lerbohe-1* and *lerboh lerbohe-1* pollens were inactive, appearing weakly stained, small, and shriveled. When observed by scanning electron microscope (SEM), many normal oval pollens were released from the dehiscent anthers of AC and *lerboh-1* while only a few shrunken pollens adhered around the uncracked anthers of *lerboh lerbohe-1* (Figure 3). Significantly, most *lerbohe-1* pollens looked the same as the double mutant, but a few ones exhibited nearly normal morphology. Thus, our data suggested that the male sterility was largely caused by abnormal pollens.

**FIGURE 4:**
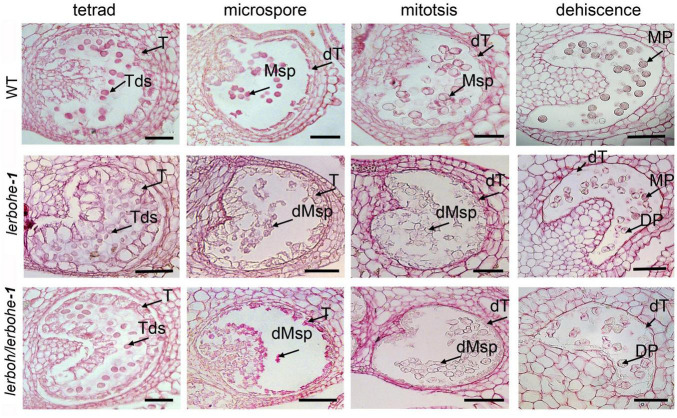
Phenotypic analysis of the tapetum layer in AC, *lerbohe* or *lerboh lerboh* anthers via paraffin sectioning. T, tapetum; dT, degraded tapetum; Tds, tetrad; PMC, pollen mother cell; Msp, microspores; MP, mature pollen; DP, degraded pollen; dMsp, degraded microspores. Scale bar: 100 μm.

To understand the specific causes for the abortive pollens, four anther developmental stages, including tetrad, microspore, mitosis, and dehiscence stages (Jeong et al., 2014), were observed through paraffin sectioning (Figure 4). No significant differences in anther structure or germ-cell morphology were observed between AC and *lerboh lerbohe-1* during the tetrad stage, when the microsporocyte undergoes meiosis to form a tetrad. At the microspore stage, AC microspores gradually vacuolized after releasing from the tetrads and the cell wall of the tapetum began to deform. However, in *lerboh lerbohe-1* anthers, the tapetum was still hypertrophic and the microspores were shrunken. After that in the mitosis stage, the wild-type cytoplasm-thickened pollens underwent mitosis and most of the cytoplasm of the tapetum cells was degraded to provide nutrients for the microspores, whereas in *lerboh lerbohe-1*, deformed pollens shrunk together and the tapetum layer was still kept thick. Finally, during the dehiscence stage, the gradually maturing wild-type pollens became full, round and the tapetum layer was completely degraded, while the mutant pollens completely shrank without accumulation of organotrophy and tapetum degradation was still not complete. Consistent with the trends of the SEM results (Figure 3), *lerbohe-1* exhibited a similar developmental alteration as the double mutant, but contained a few pollens with nearly normal morphology during the dehiscence stage. Together, these results suggest the abortive pollens were probably caused by abnormal degradation of the tapetum layer.

### Decreased Reactive Oxygen Species Accumulation and Abnormal Programmed Cell Death in *lerboh lerbohe*

**FIGURE 5:**
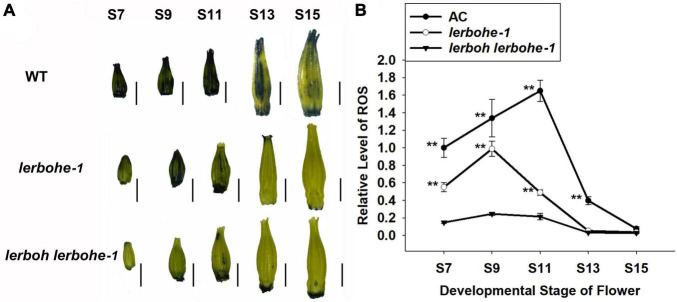
*lerbohe* and *lerboh lerbohe* anthers displayed altered ROS accumulation. **(A)** NBT staining of anthers at different developmental stages. Scale bar: 1 mm. **(B)** Quantification of relative ROS levels calculated from mean pixel densities via Image J. The data was standard by the ROS content in stage7 (S7) of AC. The statistical difference between mutant and AC lines at the same stages was analyzed by Student’s *t*-test and ** meant *P* < 0.01.

As *RBOH* genes are associated with accumulation of ROS (Xie et al., 2014), we analyzed ROS metabolism in the anthers of *lerbohe-1*, *lerboh lerbohe-1*, and AC via staining with NBT (Nitrotetrazole blue chloride), which visualizes superoxide (Figure 5 and Supplementary Figure 5). In AC, NBT staining was visible in the anthers starting at stage 7 and deepened gradually until stage 11, after which it decreased again. However, NBT staining and thus ROS content of *lerboh lerbohe* anthers was significantly lower than AC throughout anther development. The *lerbohe* mutant showed clear NBT staining through stage 11, although at reduced levels compared to WT (Figure 5). In addition, we observed hardly any differences in NBT staining between *lerboh-1* and AC (Supplementary Figure 5). These results indicated a potential role of ROS accumulation in anther development caused by *RBOH* genes.

**FIGURE 6:**
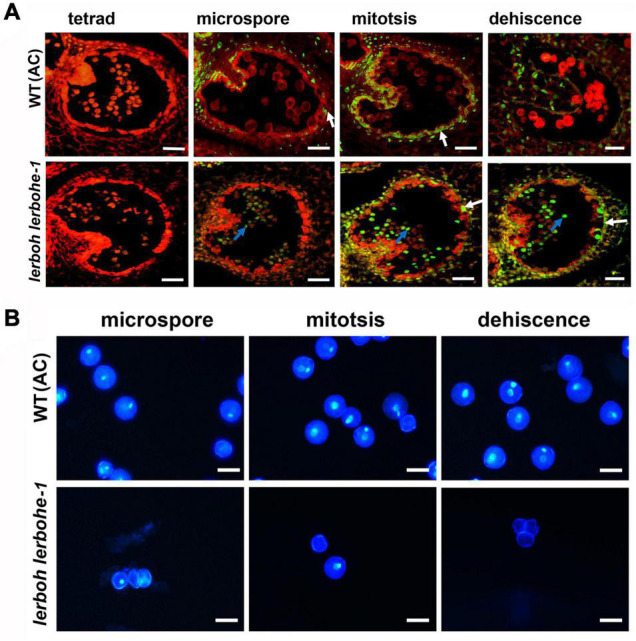
*lerboh lerbohe* anthers exhibited delayed tapetum PCD and abnormal pollen nuclei. **(A)** TUNEL analysis of anthers. Green fluorescence, TUNEL; Red fluorescence, propidium iodide staining; white arrow, degraded tapetum layers; blue arrow, degraded pollens. Scale bar: 75 μm. **(B)** DAPI staining of pollens. The bright, white dots are the pollen nuclei. Scale bar: 25 μm.

As the degradation of tapetum layers was usually a result of PCD which was also closely related to ROS metabolism, we evaluated the progression of tapetal PCD via the TUNEL (Terminal-deoxynucleotidyl-transferase-mediated dUTP Nick-End Labeling) assay in the WT and *lerboh lerbohe* lines. Since the temporal changes in ROS accumulation were not completely vanished in *lerbohe-1*, we did not further evaluate it (Figure 5). In the WT tapetum, positive TUNEL signals (indicating PCD) were visible in the microspore to dehiscence stages, consistent with the gradual degradation of tapetum cells (Joanna, 2003). In contrast, no obvious TUNEL positive signals appeared in the tapetum of the *lerboh lerbohe* mutant until the mitosis stage, but significant positive signals emerged in genital cells since the microspore stage (Figure 6A), consistent with the observed tapetum degradation delay and the inactive pollen grains (Figure 4). Furthermore, DAPI staining revealed that there were one (in microspores) or two nuclei (in mitotic or mature pollens) in AC cells. However, in *lerboh lerbohe-1* and *lerbohe-1*, only a few one-nuclei cells (microspores and mitotic pollens) were observed and nearly all mature pollens were enucleated (Figure 6B and Supplementary Figure 6). Together these results demonstrate that our male sterile strain might be caused by abnormal PCD of tapetum cells, which was perhaps associated with the changed ROS accumulation in tomato anthers.

## Discussion

### *lerbohe* and *lerboh lerbohe* Mutants Were Useful Male Sterile Strains

Cross-breeding is critical for the tomato industry as it increases fruit yield, stress tolerance and disease resistance. Male sterile lines are invaluable for this process as they significantly promote the efficiency of hybridization (Kim and Zhang, 2018). However, most existing male sterile germplasms still exhibit problematic defects, such as partial self-crossing, difficult artificial pollination, or a low cross-seed setting rate (Perez-Prat and Campagne, 2002; Chen et al., 2004). Here, by mutating two tomato anther-expressed *RBOH* genes via CRISPR/Cas9-mediated gene editing, we obtained male sterile strains with several advantages to existing lines. Firstly, pollens of either *lerbohe* or *lerboh lerbohe* mutants were completely abortive, resulting in no self-crossing (Figures 2, 3 and Supplementary Figure 4). Secondly, both *lerbohe* and *lerboh lerbohe* flowers had normal pistils (Figure 2G) and could bear normal fruits full of active seeds when given normal pollens (Figures 2H–K), indicating an easier pollination and a better outcrossing rate in cross-breeding. Thirdly, unlike many weak growing mutants which have fewer flowers, knocking out *LeRBOHE* and/or *LeRBOH* did not noticeably influence the numbers and the reproductive function of flowers, except for the abnormal maturation of anthers (Figure 2). In addition, although our mutants were strict male sterile, they could be maintained by hybridization between female parent *lerbohe* or *lerboh lerbohe* and the male parent *LeRBOHE*^±^ or *lerboh LeRBOHE^±^*, respectively, by which half F_1_ plants were identified as male sterile lines after flowering while another half were heterozygote used for the next round of hybridization. Taken together, we consider *lerbohe* and *lerboh lerbohe* mutants as useful male sterile strains for tomato hybrid breeding.

### *LeRBOHE*/*LeRBOH* Regulated Pollen Maturation via Reactive Oxygen Species Accumulation and Tapetum Programmed Cell Death

Abortive pollens are usually caused by abnormal development of the tapetum, which provides nutritional support for the maturation of microspores (Pacini, 2010). A recent study revealed that *SlMS10* (a *bHLH* transcription factor) regulates meiosis and cell death of the tapetum during microsporogenesis in tomatoes, thereby influencing pollen abortion (Yu et al., 2020). Our male sterile mutants exhibited delayed PCD in the tapetum (Figure 6), which would create a starvation stress for the microspores. As a result, we observed dying pollen cells (with abnormal nuclei numbers) and subsequently inactive pollens with weird morphology (Figures 4, 6), which demonstrated the key role of tapetum PCD in tomato pollen maturation. ROS is also regarded as a key regulator of the tapetum (Hu et al., 2011; Luo et al., 2013), and RBOH has been implicated in ROS production (Xie et al., 2014). In our work, *lerbohe* and *lerboh lerbohe* mutants displayed lighter NBT stain and delayed degradation of tapetum than WT (Figures 5, 6), revealing a positive relationship between ROS accumulation and PCD during tapetum development. Taken together, we inferred a *LeRBOHE*/*LeRBOH*-ROS-PCD pathway in regulating tomato pollen maturation.

### *LeRBOHE* and *LeRBOH* Regulated Male Sterility Unequally

In *Arabidopsis*, RBOH constitutes a protein family consisting of many conserved members which have different expression patterns and influence ROS synthesis in different tissues (Sun et al., 2015). Among the 8 tomato *RBOH* genes, *LeRBOHE* and *LeRBOH* exhibit expression peaks in the anthers, with *LeRBOH* being specifically expressed only in the anthers (Yu et al., 2017). Unexpectedly, we did not observe any abnormal phenotypes in neither the anthers nor pollens of the *lerboh* line, which suggests *LeRBOH* is not essential for male reproduction (Figure 2). Interestingly, our results revealed the key role of *LeRBOHE* in tapetum development in the anthers, as knock out of *LeRBOHE* induced male sterility. This partially agrees with the trends seen with *Arabidopsis RBOHE* (Xie et al., 2014). However, *atrbohe* displays only partial pollen sterility in contrast to the complete male sterility seen with *lerbohe*, illustrating that *RBOHE* is critical during anther development in tomato. However, the decrease in ROS content was less severe in the anthers of *lerbohe-1* than in those of the *lerboh lerbohe* double mutant (Supplementary Figure 5). Moreover, we found that there were a few morphologically normal pollens in *lerbohe-1* (Figure 3), which were hardly observed in *lerboh lerbohe* anthers. In addition, the morphologically normal pollens in *lerbohe-1* were still enucleated just as the *lerboh lerbohe* ones (Supplementary Figure 6). Together, these results indicate that there is a slight contribution of *LeRBOH* in regulating ROS synthesis and pollen development, although it has no essential effect on male sterility. Thus, we concluded that knocking out *LeRBOHE* was sufficient to generate a male sterile germplasm, but knockout of both *LeRBOH* and *LeRBOHE* rendered all pollens abnormal and non-functional.

According to the researches in many species such as Arabidopsis, rice, and tomato, some *RBOH* homologous express widely during the vegetative growth stage and are closely related to stress tolerance and disease resistance (Yoshiaki et al., 2005; Sun et al., 2015; Orman-Ligeza et al., 2016; Xu et al., 2021). Aside from the high-expression in anthers, some transcriptome data exhibits that *LeRBOH* and *LeRBOHE* also express more or less in vegetative tissues (Tomato Functional Genomics Database), which perhaps indicates their role beyond reproductive regulation. However, we observed no significant difference of vegetative growth phenotype among *lerboh*, *lerbohe*, *lerboh lerbohe*, and wild type tomatoes under normal cultivation conditions, probably due to the redundancy of other *RBOH* homologous expressed in tomato vegetative organs (Yu et al., 2017). In addition, whether *LeRBOH* and *LeRBOHE* play roles in regulating tomato stress response is still worth investigating in future.

## Conclusion

We found the function of *LeRBOHE* and *LeRBOH* in anther development, revealing the LeRBOHE/LeRBOH-ROS-PCD pathway in regulating pollen maturation of tomatoes. Additionally, the success of disruption tomato male reproduction via editing of *RBOH* genes perhaps could be used in generating male sterile germplasms for tomato hybrid breeding in the near future.

## Data Availability Statement

The original contributions presented in the study are included in the article/Supplementary Materials, further inquiries can be directed to the corresponding author/s.

## Author Contributions

XD, HH, CM, and HX conceived the project, analyzed the data, and wrote the manuscript. XD and HH designed the sgRNAs and analyzed the phenotypes of the mutants. Other authors contributed to phenotype analysis and provided important suggestions for this work. HH, CM, and HX supervised the project. All authors contributed to the article and approved the submitted version.

## Conflict of Interest

XD, CL, XN, and HX were employed by the company BellaGen Biotechnology Co., Ltd. The remaining authors declare that the research was conducted in the absence of any commercial or financial relationships that could be construed as a potential conflict of interest.

## Publisher’s Note

All claims expressed in this article are solely those of the authors and do not necessarily represent those of their affiliated organizations, or those of the publisher, the editors and the reviewers. Any product that may be evaluated in this article, or claim that may be made by its manufacturer, is not guaranteed or endorsed by the publisher.

## Footnotes

1
https://solgenomics.net/

## References

[B1] Cao X. Liu X. Wang X. Yang M. Giang T. Wang J. (2019). B-class MADS-box TM6 is a candidate gene for tomato male sterile-1526. *Theor. Appl. Genet.* 132 2125–2135. 10.1007/s00122-019-03342-z 31020387

[B2] Chapman J. M. Muhlemann J. K. Gayomba S. R. Muday G. K. (2019). RBOH-dependent ROS synthesis and ROS scavenging by plant specialized metabolites to modulate plant development and stress responses. *Chem. Res. Toxicol.* 32:3. 10.1021/acs.chemrestox.9b00028 30781949 PMC6857786

[B3] Cheema D. S. Dhaliwal M. S. (2005). Hybrid tomato breeding. *New Seeds* 6 1–14. 10.1300/J153v06n02_01

[B4] Chen Y. H. Xu X. Y. Li G. Y. (2004). Review of advances in research of the male sterility in tomato. *J. Northeast Agric. Univ.* 35 129–134. 10.1007/BF02873091

[B5] Du M. Zhou K. Liu Y. Deng L. Zhang X. Lin L. (2020). A biotechnology-based male-sterility system for hybrid seed production in tomato. *Plant J.* 102 1090–1100. 10.1111/tpj.14678 31923323 PMC7317546

[B6] Fu Z. Yu J. Cheng X. Zong X. Xu J. Chen M. (2014). The rice basic helix-loop-helix transcription factor TDR INTERACTING PROTEIN2 is a central switch in early anther development. *Plant Cell* 26 1512–1524. 10.1105/tpc.114.123745 24755456 PMC4036568

[B7] Gorguet B. Schipper D. Lammeren A. Visser R. G. Heusden A. W. (2009). PS-2, the gene responsible for functional sterility in tomato, due to non-dehiscent anthers, is the result of a mutation in a novel polygalacturonase gene. *Theor. Appl. Genet.* 118 1199–1209. 10.1007/s00122-009-0974-9 19219598

[B8] Gorman S. W. McCormick S. Rick C. (1997). Male sterility in tomato. *Plant Sci.* 16 31–53. 10.1080/07352689709701945

[B9] Hu L. Liang W. Yin C. Cui X. Zong J. Wang X. (2011). Rice MADS3 regulates ROS homeostasis during late anther development. *Plant Cell* 23 515–533. 10.1105/tpc.110.074369 21297036 PMC3077785

[B10] Jeong H. J. Kang J. H. Zhao M. Kwon J. K. Choi H. S. Bae J. H. (2014). Tomato male sterile 1035 is essential for pollen development and meiosis in anthers. *J. Exp. Bot.* 65:6693. 10.1093/jxb/eru389 25262227 PMC4246194

[B11] Joanna L. (2003). Anther tapetum in programmed cell death. *Kosmos* 52 99–412.

[B12] Kim Y. J. Zhang D. B. (2018). Molecular control of male fertility for crop hybrid breeding. *Trends Plant Sci.* 23 53–65. 10.1016/j.tplants.2017.10.001 29126789

[B13] Li N. Zhang D. S. Liu H. S. Yin C. S. Li X. X. Liang W. Q. (2006). The rice tapetum degeneration retardation gene is required for tapetum degradation and anther development. *Plant Cell* 18 2999–3014. 10.1105/tpc.106.044107 17138695 PMC1693939

[B14] Liu X. Yang M. Liu X. Wei K. Cao X. Wang X. (2019). A putative bHLH transcription factor is a candidate gene for male sterile 32, a locus affecting pollen and tapetum development in tomato. *Hortic. Res. Engl.* 6:88. 10.1038/s41438-019-0170-2 31666957 PMC6804878

[B15] Luo D. Xu H. Liu Z. Guo J. Li H. Chen L. (2013). A detrimental mitochondrial-nuclear interaction causes cytoplasmic male sterility in rice. *Nat. Genet.* 45 573–577. 10.1038/ng.2570 23502780

[B16] Orman-Ligeza B. Parizot B. Rycke R. D. Fernandez A. Himschoot E. Breusegem F. V. (2016). RBOH-mediated ROS production facilitates lateral root emergence in *Arabidopsis*. *Development* 143 3328–3339. 10.1242/dev.136465 27402709 PMC5047660

[B17] Pacini E. (2010). Relationships between tapetum, loculus, and pollen during development. *Int. J. Plant Sci.* 171 1–11. 10.1086/647923

[B18] Peng Z. Cheng L. He Y. J. Wang J. Guan X. Y. Liu S. Y. (2013). Cytological study on microsporogenesis of *Solanum lycopersicum* var. Micro-Tom under high temperature stress. *Acta Ecol. Sin.* 33 2084–2092. 10.5846/stxb201112261972

[B19] Perez-Prat E. Campagne M. M. V. L. (2002). Hybrid seed production and the challenge of propagating male-sterile plants. *Trends Plant Sci.* 7 199–203. 10.1016/S1360-1385(02)02252-5 11992824

[B20] Phan H. A. Iacuone S. Li F. S. Parish R. W. (2011). The MYB80 transcription factor is required for pollen development and the regulation of tapetal programmed cell death in *Arabidopsis thaliana*. *Plant Cell* 23 2209–2224. 10.1105/tpc.110.082651 21673079 PMC3160043

[B21] Porebski S. Bailey L. G. Baum B. R. (1997). Modification of a CTAB DNA extraction protocol for plants containing high polysaccharide and polyphenol components. *Plant Mol. Bio. Rep.* 15 8–15. 10.1007/BF02772108

[B22] Pucci A. Picarella M. E. Mazzucato A. (2017). Phenotypic, genetic and molecular characterization of 7B-1, a conditional male-sterile mutant in tomato. *Theor. Appl. Genet.* 130 2361–2374. 10.1007/s00122-017-2964-7 28815278

[B23] Sawhney V. K. (2004). Photoperiod-sensitive male-sterile mutant in tomato and its potential use in hybrid seed production. *J. Hortic. Sci. Biotechnol.* 79 138–141. 10.1080/14620316.2004.11511726

[B24] Shen L. Dong G. Zhang Y. Hu G. Zhan Q. Hu G. (2019). Rapid creation of new photoperiod-/thermo-sensitive genic male-sterile rice materials by CRISPR/Cas9 system. *Rice Sci.* 26 129–132. 10.1016/j.rsci.2018.12.006

[B25] Sun X. Hu X. Yang Y. (2015). Molecular and functional comparisons of reactive burst oxygen species gene family in *Arabidopsis*. *Plant Diversity Resour.* 4 463–471.

[B26] Tamta S. Singh J. P. (2017). Heterosis in tomato for growth and yield traits. *Int. J. Veg. Sci.* 24 169–179. 10.1080/19315260.2017.1407857

[B27] Ullah F. Yi M. (2019). Mechanisms of ROS regulation of plant development and stress responses. *Front. Plant Sci.* 10:800. 10.3389/fpls.2019.00800 31293607 PMC6603150

[B28] Wang X. Guan Z. Gong Z. Yan J. Yang G. Liu Y. (2018). Crystal structure of WA352 provides insight into cytoplasmic male sterility in rice. *Biochem. Biophys. Res. Commun.* 501 898–904. 10.1016/j.bbrc.2018.05.079 29775612

[B29] Xie H. T. Wan Z. Y. Li S. Zhang Y. (2014). Spatiotemporal production of reactive oxygen species by NADPH oxidase is critical for tapetal programmed cell death and pollen development in *Arabidopsis*. *Plant Cell* 26 2007–2023. 10.1105/tpc.114.125427 24808050 PMC4079365

[B30] Xu J. Kang Z. Zhu K. Zhao D. Yuan Y. Yang S. (2021). RBOH1-dependent H2O2 mediates spermine-induced antioxidant enzyme system to enhance tomato seedling tolerance to salinity-alkalinity stress. *Plant Physiol. Biochem.* 164:2. 10.1016/j.plaphy.2021.04.017 34015689

[B31] Yan M. Y. Xie D. L. Cao J. J. Xia X. J. Shi K. Zhou Y. H. (2020). Brassinosteroid mediated reactive oxygen species are essential for tapetum degradation and pollen fertility in tomato. *Plant J.* 102 931–947. 10.1111/tpj.14672 31908046

[B32] Yi J. Moon S. Lee Y. Zhu L. Liang W. Zhang D. (2016). Defective tapetum cell death 1 (DTC1) regulates ROS levels by binding to metallothionein during tapetum degeneration. *Plant Physiol.* 170 1611–1623. 10.1104/pp.15.01561 26697896 PMC4775127

[B33] Yoshiaki Y. Kazunori G. Ryota T. Megumi I. Seiji T. Akiraet I. (2005). Function of the rice gp91phox homologs OsrbohA and OsrbohE genes in ROS-dependent plant immune responses. *Plant Biotechnol.* 22 127–135. 10.5511/plantbiotechnology.22.127

[B34] Yu J. J. Dong H. K. Lee H. J. Nam K. H. Bae S. Nou I. S. (2020). Knockout of SlMS10 gene (Solyc02g079810) encoding bHLH transcription factor using CRISPR/Cas9 system confers male sterility phenotype in tomato. *Plants* 9:1189. 10.3390/plants9091189 32933074 PMC7570381

[B35] Yu S. X. Feng Q. N. Xie H. T. Li S. Zhang Y. (2017). Reactive oxygen species mediate tapetal programmed cell death in tobacco and tomato. *BMC Plant Biol.* 17:76. 10.1186/s12870-017-1025-3 28427341 PMC5399379

[B36] Zhang L. Huang Z. Wang X. Gao J. Guo Y. Du Y. (2016). Fine mapping and molecular marker development of anthocyanin absent, a seedling morphological marker for the selection of male sterile 10 in tomato. *Mol. Breed.* 36:107.

[B37] Zhu E. You C. Wang S. Jie C. Niu B. Wang Y. (2015). The DYT1-interacting proteins bHLH010, bHLH089 and bHLH091 are redundantly required for *Arabidopsis* anther development and transcriptome. *Plant J.* 83 976–990. 10.1111/tpj.12942 26216374

