# Supplementary material for: Generating Novel Male Sterile Tomatoes by Editing Respiratory Burst Oxidase Homolog Genes
Source: Front Plant Sci. 2022 Jan 10;12:817101. doi: 10.3389/fpls.2021.817101 (PMC8784783; doi:10.3389/fpls.2021.817101)
Supplement: Supplementary file 1 [file Data_Sheet_1.PDF]

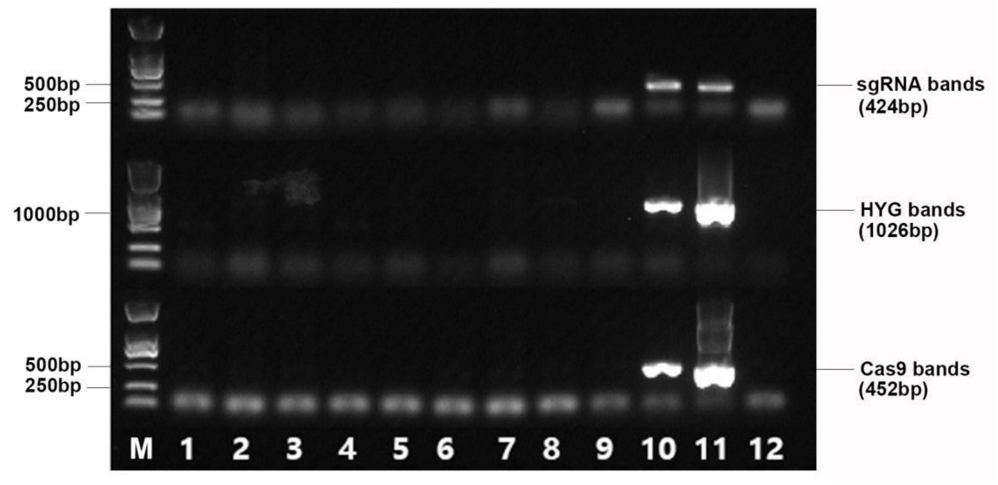

Figure S1. Identification of T-DNA free editing mutants (T_1). Lanes1, *lerboh*; 2, *lerbohe-1*; 3, *lerbohe-2*; 4, *lerboh lerbohe-1*; 5, *lerboh lerbohe-2*; 6-8 *lerboh LeRBOHE*$^{+/-}$; 9, WT(AC); 10, T-DNA insertion positive line; 11, vector control; 12, negative control.

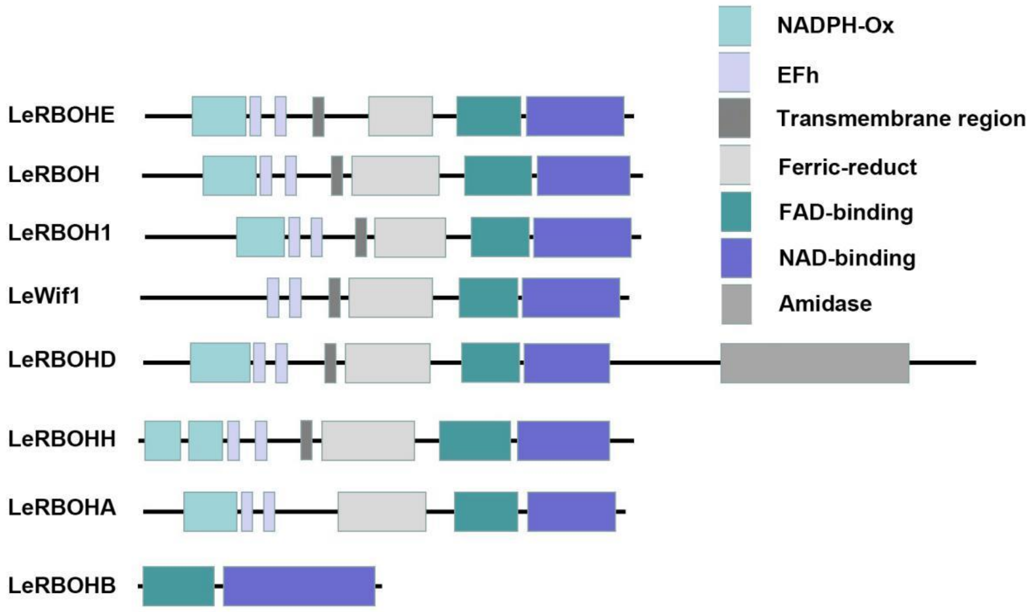

Figure S2. Structure domain analysis of LeRBOHs. The main structure domains in LeRBOHs were identified by SMART domain analysis (http://smart.embl-heidelberg.de) and displayed as rectangular bars.

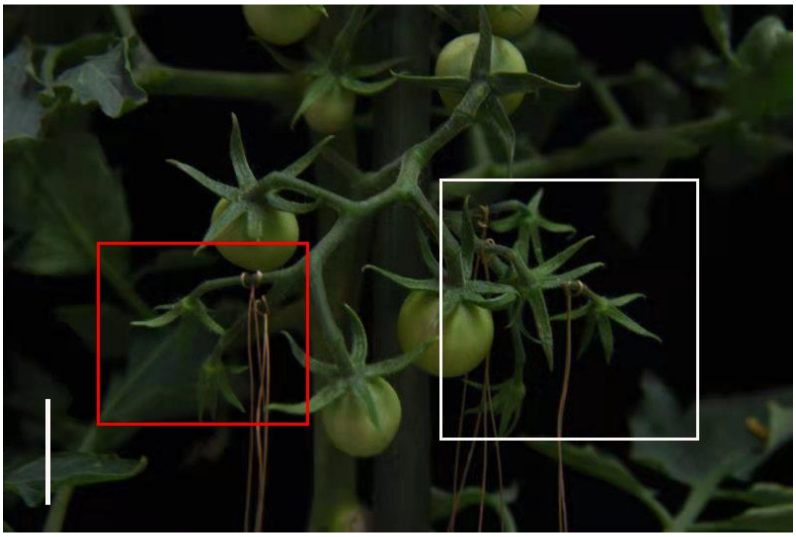

Figure S3. Pollination for AC pistils with pollens of *lerbohe-1* or *lerboh lerbohe-1*. The emasculated AC pistils were pollinated by *lerbohe* pollens (marked by red frame) or *lerboh lerbohe* pollens (marked by white frame). Scale bar: 2 cm.

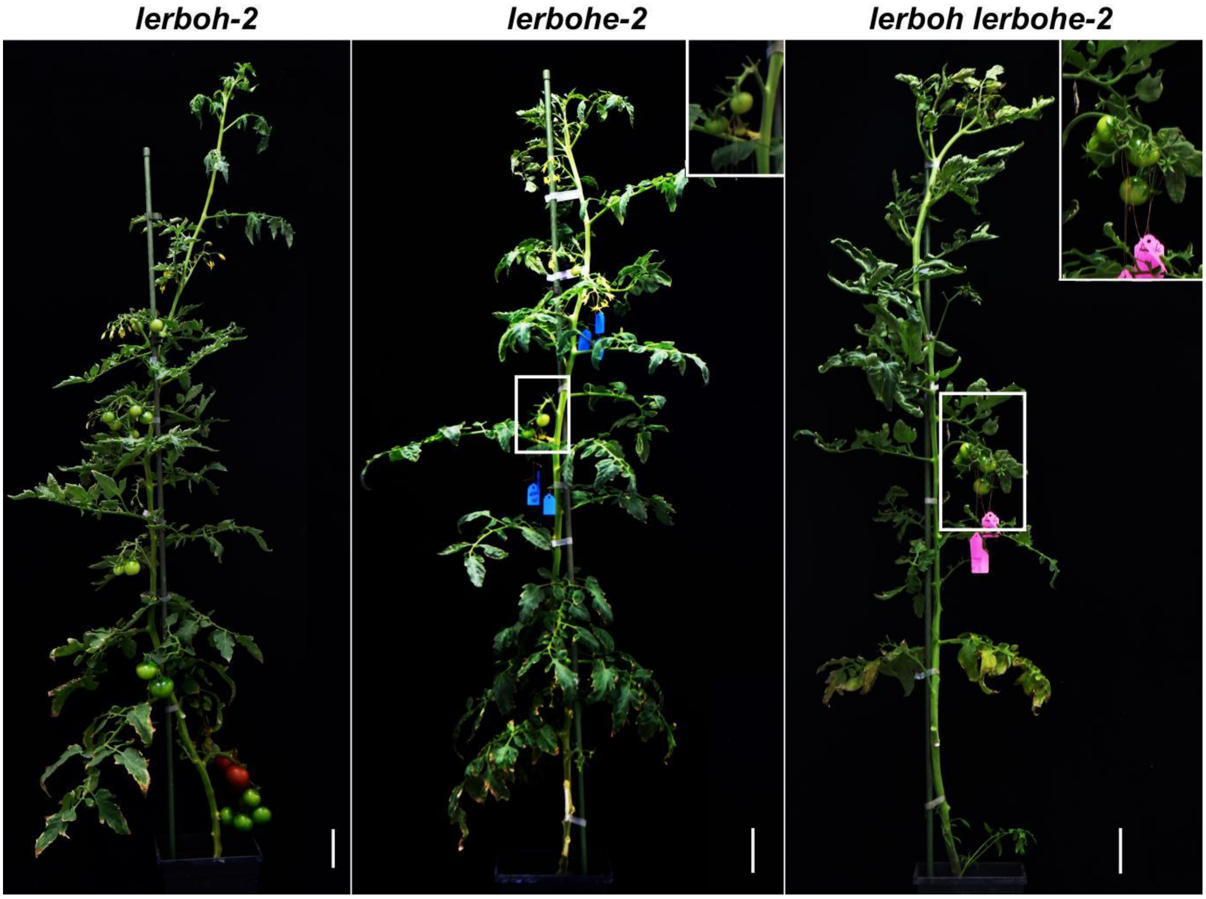

Figure S4. Phenotypic analysis of *lerboh-2*, *lerbohe-2* and *lerboh lerbohe-2*. Flowers and fruits within tags were fertilized by AC pollens (in white frames). Scale bar: 5 cm.

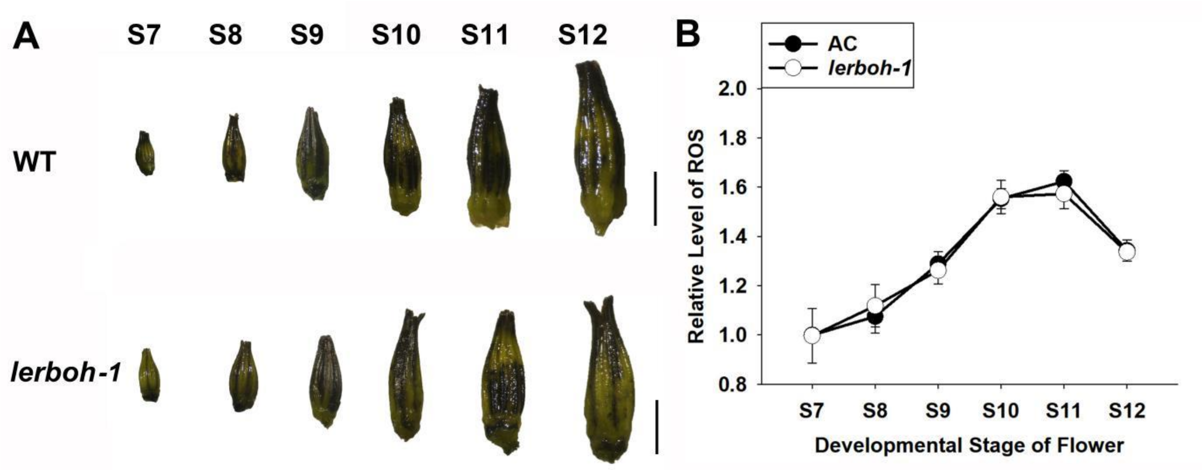

Figure S5. Analysis of ROS accumulation in *lerboh* and AC anthers. (A) NBT staining of anthers at different developmental stages. Scale bar: 1 mm. (B) Quantification of relative ROS levels calculated from mean pixel densities via Image J. Data was analyzed by student's t test and there was no significant difference between mutant and AC lines at the same stages.

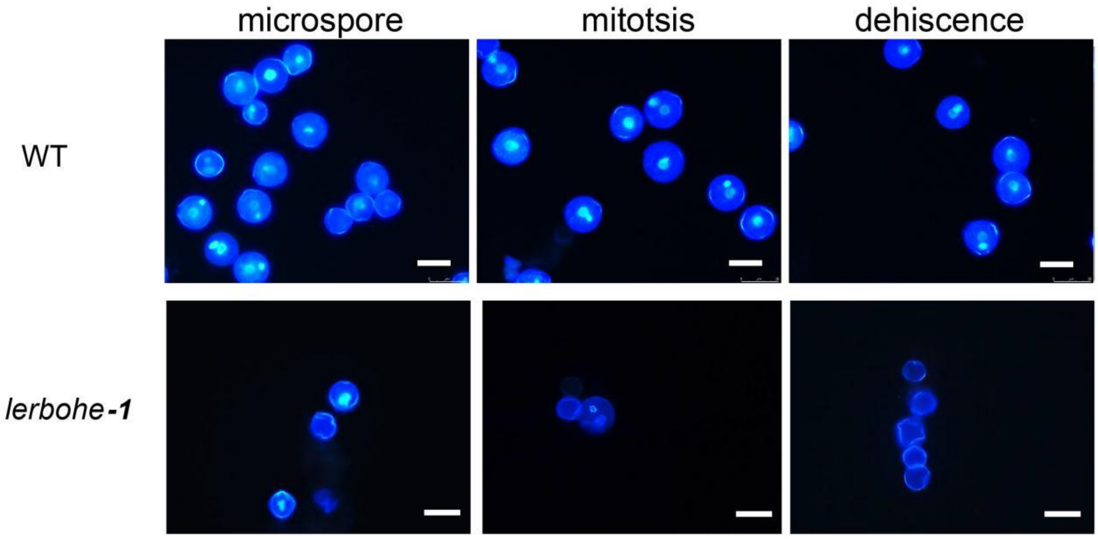

Figure S6. DAPI staining of AC and *lerbohe-1* pollens. The white brightened dots are pollen nuclei. Scale bar: 25 μm.

Table S1. Primers used in this work.

Primer name	Primer sequences（5'-3'）
Cas9-F	AAGAAGCGGAAGGTCGGTAT
Cas9-R	CTCAGGTGGTAGATGGTGGG
M13F	TGTAAAACGACGGCCAGT
gRNA-R	CTAAAAACAAGACGGATTACTTGCTAGA
Hyg-F	ATGAAAAAGCCTGAACTCACC
Hyg-R	CTATTTCTTTGCCCTCGGAC
LeRBOH-F	TGTGAGAGGATCGAGAGTAGG
LeRBOH-R	TAGTCACCGCAGGAGATGTG
LeRBOHE-F	AGCAGCATCGGAAGTGTCG
LeRBOHE-R	AGAGAAACCCACATGTCAAAGAA
gLeRBOHE-F	CGATTCCCGGCTGGTGCATCTAGCAAGTAATC
	CGTCTTGTTTAGAGCAGATAGAAATAGCAAGTT
gLeRBOHE-R	AACTTGCTATTTCTAGCTCTAAAACCTTTTCGA
	GCTTGCTAGCTATACACCA GAAGGGAATCG
